# Supplementary material for: ProMeta: a meta-learning framework for robust disease diagnosis and prediction from plasma proteomics
Source: Bioinformatics. 2026 Jul 7;42(Suppl 1):btag267. doi: 10.1093/bioinformatics/btag267 (PMC13340251; doi:10.1093/bioinformatics/btag267)
Supplement: btag267_Supplementary_Data [file btag267_supplementary_data.pdf]

Supplemental Methods

Pseudocode of ProMeta

Algorithm 1: ProMeta Meta-Training Algorithm

Input: Task distribution $P(\mathcal{T})$, Pathway mask $\mathbf{M}$, Inner steps K, Outer learning rate α, Inner learning rate β

Output: Optimized initial model parameters Θ

Initialize global meta-parameters Θ;
while *not converged* **do**
    Sample a batch of tasks $\mathcal{B} \sim P(\mathcal{T})$;
    for *each task* $\mathcal{T}_i \in \mathcal{B}$ **do**
        Sample support set $\mathcal{S}_i = (\mathbf{x}_s, y_s)$ and query set $\mathcal{Q}_i = (\mathbf{x}_q, y_q)$ from $\mathcal{T}_i$;
        Initialize task-adapted parameters $\Phi_i \leftarrow \Theta$;
        `// Inner loop: Task-specific adaptation`
        for $k = 1$ **to** K **do**
            `// Forward pass` f `embeds PathwayGatedTokenizer (`g`), MetaTransformer (`h`) and Classifier (`c`)`
            Evaluate $\mathcal{L}_{\mathcal{S}_i} =$ FocalLoss$(f(\mathbf{x}_s; \Phi_i, \mathbf{M}), y_s)$;
            Update adapted parameters: $\Phi'_i \leftarrow \Phi_i - \beta \nabla_{\Phi_i} \mathcal{L}_{\mathcal{S}_i}$;
        `// Evaluate meta-objective on query set`
        Compute $\mathcal{L}_{\mathcal{Q}_i} =$ FocalLoss$(f(\mathbf{x}_q; \Phi'_i, \mathbf{M}), y_q)$;
    `// Outer loop: Global meta-update`
    Update global model: $(\Theta, \beta) \leftarrow (\Theta, \beta) - \eta \nabla_{(\Phi,\beta)} \sum_{\mathcal{T}_i} \mathcal{L}_{\mathcal{Q}_i}(\Phi'_i)$;
return Θ

Algorithm 2: ProMeta Meta-Testing Procedure

Input: Trained meta-parameters Θ, Test task distribution $P(\mathcal{T})$, Pathway mask $\mathbf{M}$, Inner update steps K, Inner learning rate β

Output: Mean performance metrics across test tasks

Initialize metric accumulator set $\mathcal{A} \leftarrow \emptyset$;

Let $\Theta_{\text{adapt}} \subset \Theta$ be the subset of adaptable parameters (soft input filter θ and the final linear classifier W_{clf}, b_{clf});

for *each test task* $\mathcal{T}_j \sim P(\mathcal{T})$ **do**

- Sample support set $\mathcal{S}_j$ and unseen query set $\mathcal{Q}_j$ from $\mathcal{T}_j$;
- Initialize task-adapted parameters $\Phi_j \leftarrow \Theta$;
- `// Adaptation: Fine-tune on Support Set`
- **for** $k = 1$ **to** K **do**
  - Evaluate loss: $\mathcal{L}_{\mathcal{S}_j} = \text{FocalLoss}(f(\mathcal{S}_j; \Phi_j, \mathbf{M}))$;
  - Update adaptable parameters: $\Phi_{j,\text{adapt}} \leftarrow \Phi_{j,\text{adapt}} - \beta \nabla_{\Phi_{j,\text{adapt}}} \mathcal{L}_{\mathcal{S}_j}$;
- `// Evaluation: Test on Query Set`
- Generate predictions: $\hat{y}_q = f(\mathcal{Q}_j; \Phi_j, \mathbf{M})$;
- Compute metrics (e.g., AUROC, AUPRC) using $\hat{y}_q$ and ground truth of $\mathcal{Q}_j$;
- Append results to $\mathcal{A}$;

return *Mean($\mathcal{A}$)*

Implementation details

ProMeta was implemented in PyTorch 2.6.0 with Python 3.10.12. We implemented a two-layer transformer encoder with an embedding dimension of 64 and two attention heads. The objective function incorporated a Focal loss (**?**) ($\alpha = 0.75, \gamma = 2.0$) and a sparsity regularization coefficient (λ) of 1e-3. We utilized a Meta-SGD (**?**) optimization framework where the inner loop adaptation was performed for five steps with an initial learning rate of 0.005. An Adam optimizer with a global (outer) learning rate of 1e-4 was used to train the model. The training was performed with a batch size of four for a maximum of 100 epochs, employing early stopping with a patience of 10 epochs. The experiments were carried out using an NVIDIA A800 GPU.

Baseline methods

L1-Regularized Logistic Regression (L1Logistic). L1Logistic regression was executed using the liblinear solver in scikit-learn (1.4.2). The model was configured with a regularization strength (implemented as inverse regularization strength), a maximum of 1,000 iterations to ensure convergence. For each test disease, L1Logistic was trained on the support set and evaluated on the query set.

Multi-Layer Perceptron (MLP). The MLP model was constructed using PyTorch (2.6.0) as a two-layer network (Hidden dims: 128, 32) incorporating Layer Normalization and ReLU activations to learn task-specific representations from the support set. Key hyperparameters included a learning rate of using the Adam optimizer, a dropout rate of 0.2, and a training duration of 100 epochs per task. For each test disease, MLP was trained on the support set and evaluated on the query set.

Random Forest. Random Forest classification was performed using scikit-learn (1.4.2)'s ensemble implementation. The model was instantiation with 300 estimators and "balanced" class

weights to address sample imbalance within the support sets. For each test disease, Random Forest was trained on the support set and evaluated on the query set.

XGBoost classification was executed using the XGBClassifier (xgboost library 3.1.2) (**?**) to perform gradient-boosted decision tree learning optimized with logarithmic loss. The model hyper-parameters were tuned to prevent overfitting on few-shot tasks, utilizing 40 estimators, a maximum depth of 5, and a learning rate of 0.05. For each test disease, XGBoost was trained on the support set and evaluated on the query set.

Multitask learning of Transformer (Multitask_Transformer). The Multitask Learning baseline was implemented using a TransformerEncoder architecture in PyTorch (2.6.0). The model employs a linear projection to map protein features to a 64-dimensional hidden space, followed by two Transformer encoder layers (2 heads each) to capture feature interactions, and finally branches into task-specific prediction heads sharing a common underlying representation. Training was performed on the aggregated support sets of all tasks for 50 epochs using the AdamW optimizer with a learning rate of 0.0001, employing a masked Binary Cross-Entropy loss to update only the relevant task heads for each sample. To ensure robust generalization, a dropout probability of 0.15 was applied within the Transformer layers, and gradients were computed over global batches of size 64. Multitask_Transformer was train on all the samples from the support sets of all test diseases. Multitask_Transformer was trained on all samples from the support sets of all test diseases. During training, the labels for query-set samples from the test diseases were masked to avoid information leakage. After multitask training, the model was evaluated on the query set of each disease.

Task-similarity pretraining of Transformer (TaskSimilarity_Transformer). TaskSimilarity_Transformer leverages a transfer learning strategy where a Transformer-based predictor is first pretrained on a high-resource source task most similar to the target disease before fine-tuning. All source tasks were drawn from the training diseases. Task similarity was quantified using the Jaccard index between the associated patient cohort lists, selecting the training task with the highest overlap for each test disease. The model architecture comprises a linear projection to 64 dimensions and two Transformer encoder layers (2 heads). The two-stage optimization process involved 50 epochs of pretraining on the combined support and query sets of the selected source task (learning rate 0.0003) followed by 50 epochs of fine-tuning on the target task's support set (learning rate 0.0001), both using the AdamW optimizer and binary cross-entropy loss. The source tasks were from the training diseases.

Self-supervised pre-training of Transformer (Self-supervis
ed_Transformer). The Self-supervised_Transformer was implemented using a BERT-like pre-training strategy to learn generalizable protein representations from unlabeled data before few-shot fine-tuning with 2 encoder layers (64 hidden dimensions) was pretrained to reconstruct randomly masked protein values (15% masking ratio) using Mean Squared Error (MSE) loss. Optimization was performed using the AdamW optimizer (learning rate 0.0003) for up to 200 epochs with early stopping based on validation loss. The pre-training was performed on all samples from training diseases. During the meta-testing phase, the pretrained encoder was transferred to each disease task and fine-tuned on the small support set for 50 epochs (learning rate 0.0001) to perform binary classification.

Supervised multitask pre-training of Transformer (Supervised_Tra
nsformer). The Supervised_Transformer employs a two-stage transfer learning strategy where the feature extractor is first learned via large-scale multitask classification before adapting to specific few-shot diseases. A TransformerEncoder (2 layers, 64 hidden dimensions) was initially pretrained for 10 epochs on the aggregate of all training tasks, optimizing a masked binary cross-entropy

loss with a learning rate of 0.0001 using the AdamW optimizer. This pretrained backbone was then transferred to the meta-testing phase, where it was fine-tuned for another 10 epochs on each target task's support set to specialize the prediction head for the specific disease.

Gradient-based attribution analysis

To identify the input and intermediate features that play a crucial role in ProMeta prediction, we introduce a hierarchical attribution framework tailored for meta-learning. Unlike standard interpretation methods that operate on static weights, our approach interprets the ProMeta model after it has dynamically adapted to a specific disease task. This allows us to dissect the decision-making process at two distinct levels: specific protein biomarkers (protein level) and biological mechanisms (pathway level).

Prior to gradient-based attribution analysis, we perform the inner-loop adaptation to align the ProMeta's decision boundary with the target disease $\mathcal{T}_i$ and generate task-adapted weights ϕ_i' that encode the disease-specific logic.

To identify important protein biomarkers for a specific disease task $\mathcal{T}_i$, we compute importance scores on the query set $\mathcal{Q}_i$ relative to the raw inputs. The attribution score $A_{\text{feat}}^{(i)}$ for a protein feature x is calculated as the element-wise product of the input and its gradient with respect to the predicted probability y, averaged over all samples in $\mathcal{Q}_i$:

$$A_{\text{feat}}^{(i)} = \frac{1}{|\mathcal{Q}_i|} \sum_{x \in \mathcal{Q}_i} \left(x \odot \nabla_x F(x; \phi_i') \right), \tag{1}$$

where ϕ_i' denotes the adapted weights for the target disease.

To identify biological pathways highly associated with the target disease, we integrate gradient-based attribution analysis with ProMeta's tokenizer, which projects protein inputs into latent embeddings aligned with KEGG pathways. We perform a forward pass to extract the latent pathway embeddings $\mathbf{Z} \in \mathbb{R}^{N \times D}$ (where N is the number of pathways and D is the embedding dimension) and retain the gradients $\nabla_{\mathbf{Z}}$ by backpropagating from the output. The importance of the k-th pathway is quantified using the Gradient $\times$ Activation method:

$$A_{\text{path}}^{(k)} = \sum_{d=1}^{D} \left(\nabla_{\mathbf{Z}_{k,d}} \cdot \mathbf{Z}_{k,d} \right). \tag{2}$$

By aggregating contributions across the embedding dimension D, this metric reveals whether the activation of a specific biological pathway positively or negatively influences the final diagnosis.

To mitigate confounding effects arising from sex-specific variation and systemic background noise, a set of proteins associated with sex hormones (FSHB, PAEP, INSL3, RLN2, OXT, GH2, CGB, LHB, TSHB, PRL, CSH1, and CSH2) and high-abundance secreted factors (GAST and FOLR3) were excluded from downstream analysis.

101 # Supplemental Figures

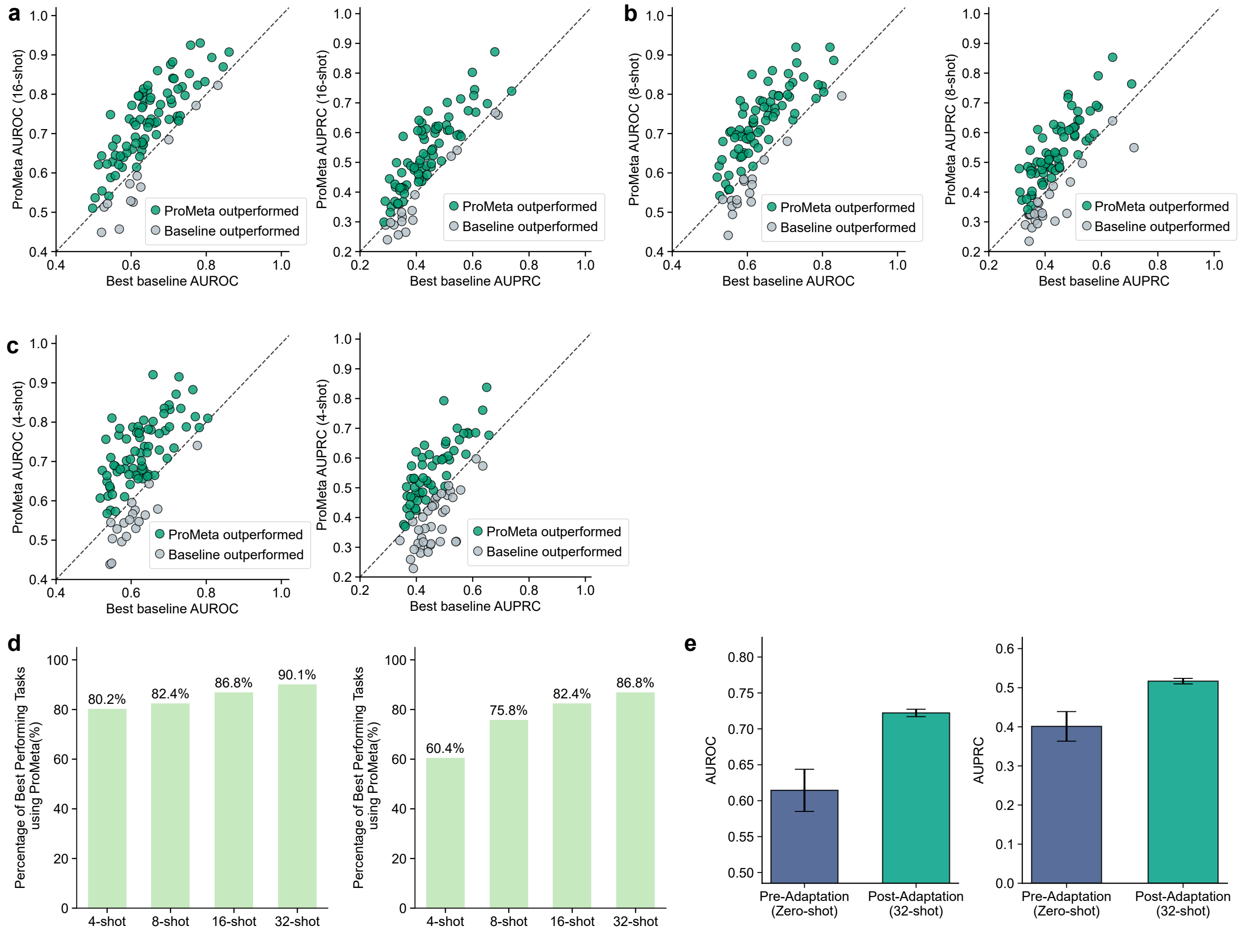

Supplementary Fig. 1: Performance evaluation for disease diagnosis in few-shot scenarios. (a–c) Head-to-head comparison of AUROC (left) and AUPRC (right) scores between ProMeta and the best-performing baseline method for each specific disease, evaluated under (a) 16-shot, (b) 8-shot, and (c) 4-shot settings. Each dot represents a unique disease endpoint. Red dots denote cases where ProMeta outperformed the best baseline method, while gray dots indicate cases where the best baseline outperformed ProMeta. (d) Win-rate analysis showing the percentage of diseases where ProMeta outperformed the best baseline method in terms of AUROC (left) and AUPRC (right) across 4-, 8-, 16-, and 32-shot scenarios. (e) Performance comparison of ProMeta prior to adaptation versus after adaptation under the 32-shot setting, measured by AUROC (left) and AUPRC (right). The bar plot and error bars denote the mean and standard deviation, respectively, calculated across five independent experimental runs with different random seeds.

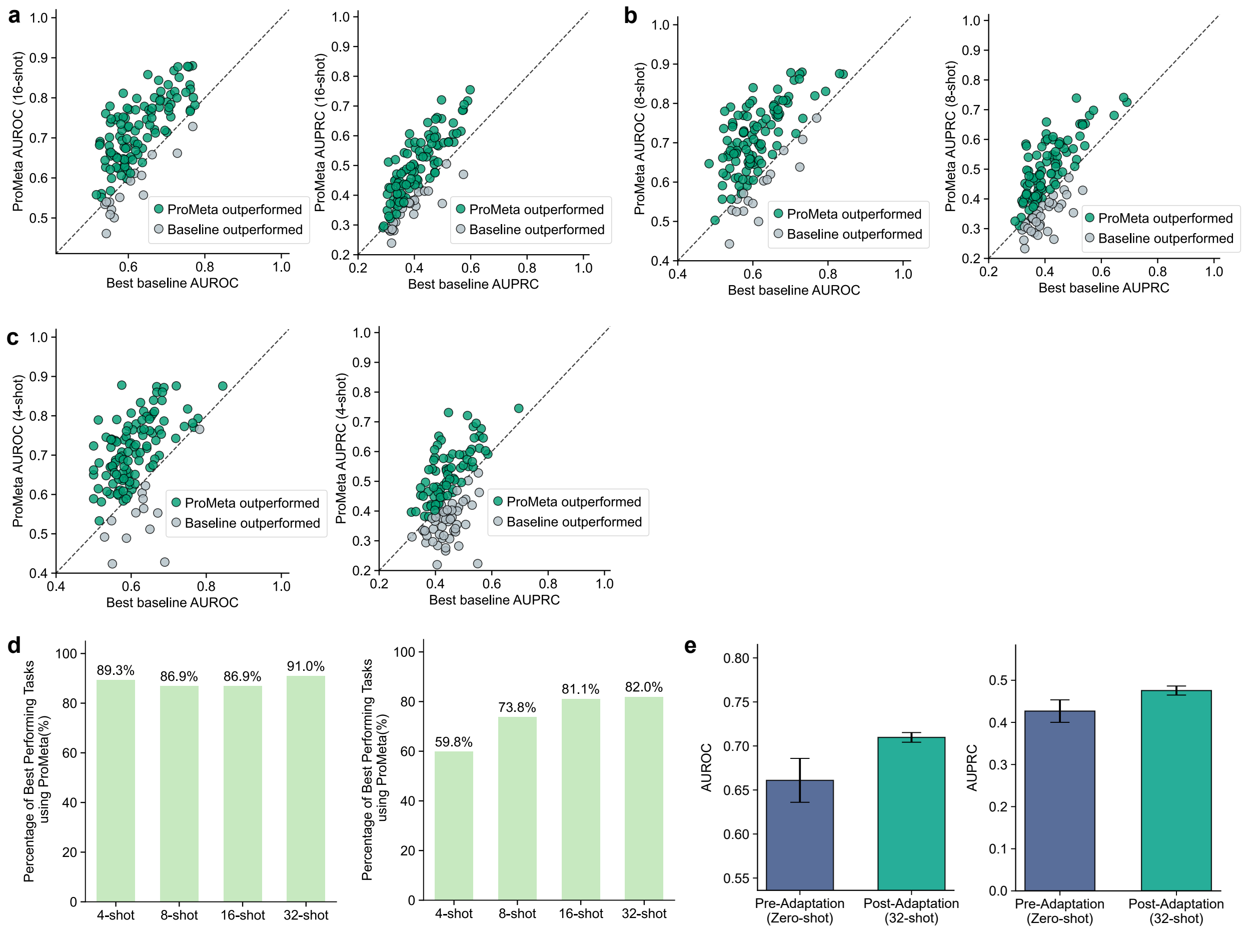

Supplementary Fig. 2: Performance evaluation for disease prediction in few-shot scenarios. (a–c) Head-to-head comparison of AUROC (left) and AUPRC (right) scores between ProMeta and the best-performing baseline method for each specific disease, evaluated under (a) 16-shot, (b) 8-shot, and (c) 4-shot settings. Each dot represents a unique disease endpoint. Red dots denote cases where ProMeta outperformed the best baseline method, while gray dots indicate cases where the best baseline outperformed ProMeta. (d) Win-rate analysis showing the percentage of diseases where ProMeta outperformed the best baseline method in terms of AUROC (left) and AUPRC (right) across 4-, 8-, 16-, and 32-shot scenarios. (e) Performance comparison of ProMeta prior to adaptation versus after adaptation under the 32-shot setting, measured by AUROC (left) and AUPRC (right). The bar plot and error bars denote the mean and standard deviation, respectively, calculated across five independent experimental runs with different random seeds.

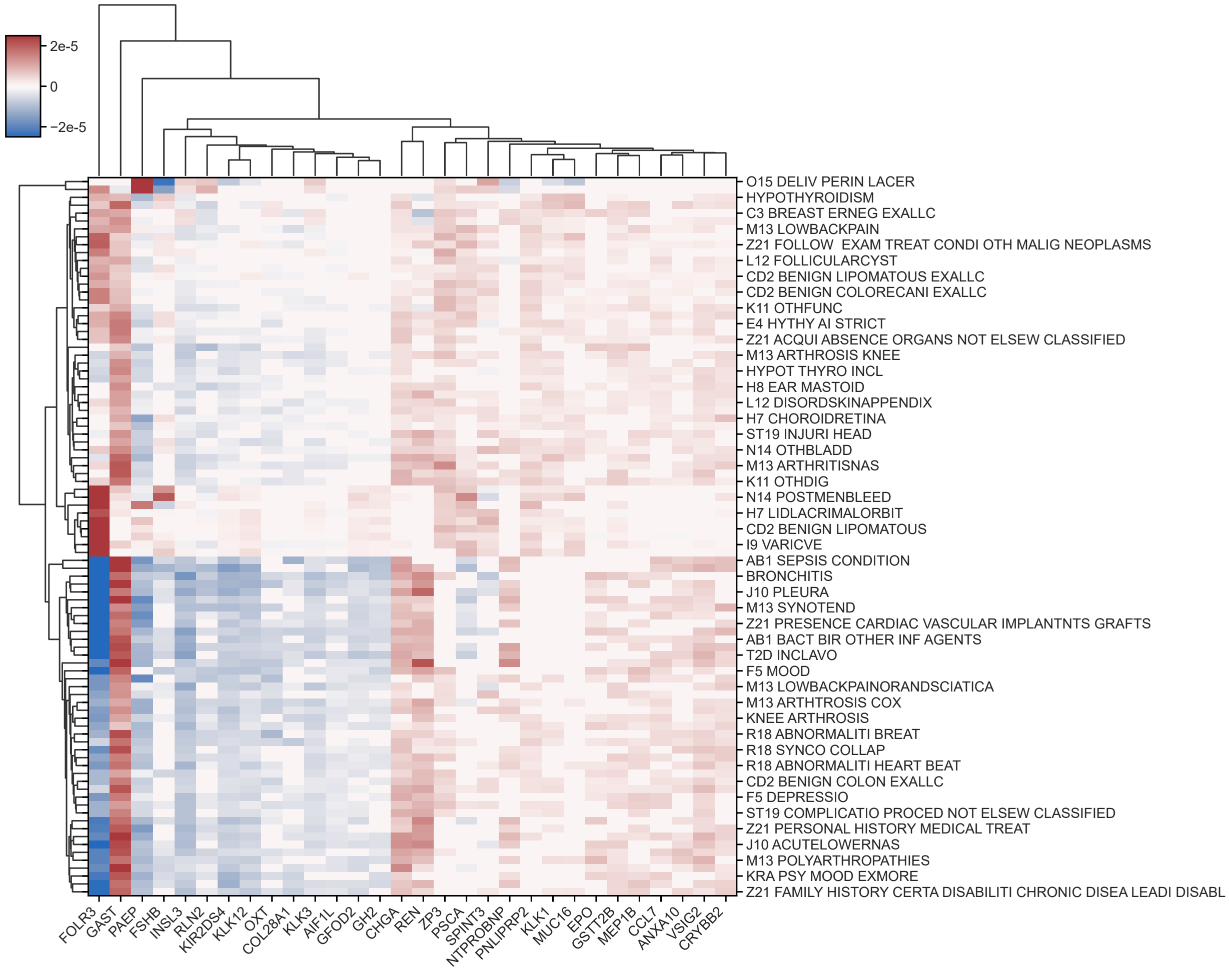

Supplementary Fig. 3: High-frequency protein biomarkers identified by ProMeta for disease diagnosis. Hierarchical clustering of these top 30 proteins across diverse disease endpoints. The heatmap visualizes the gradient-based importance scores (red: positive association; blue: negative association).

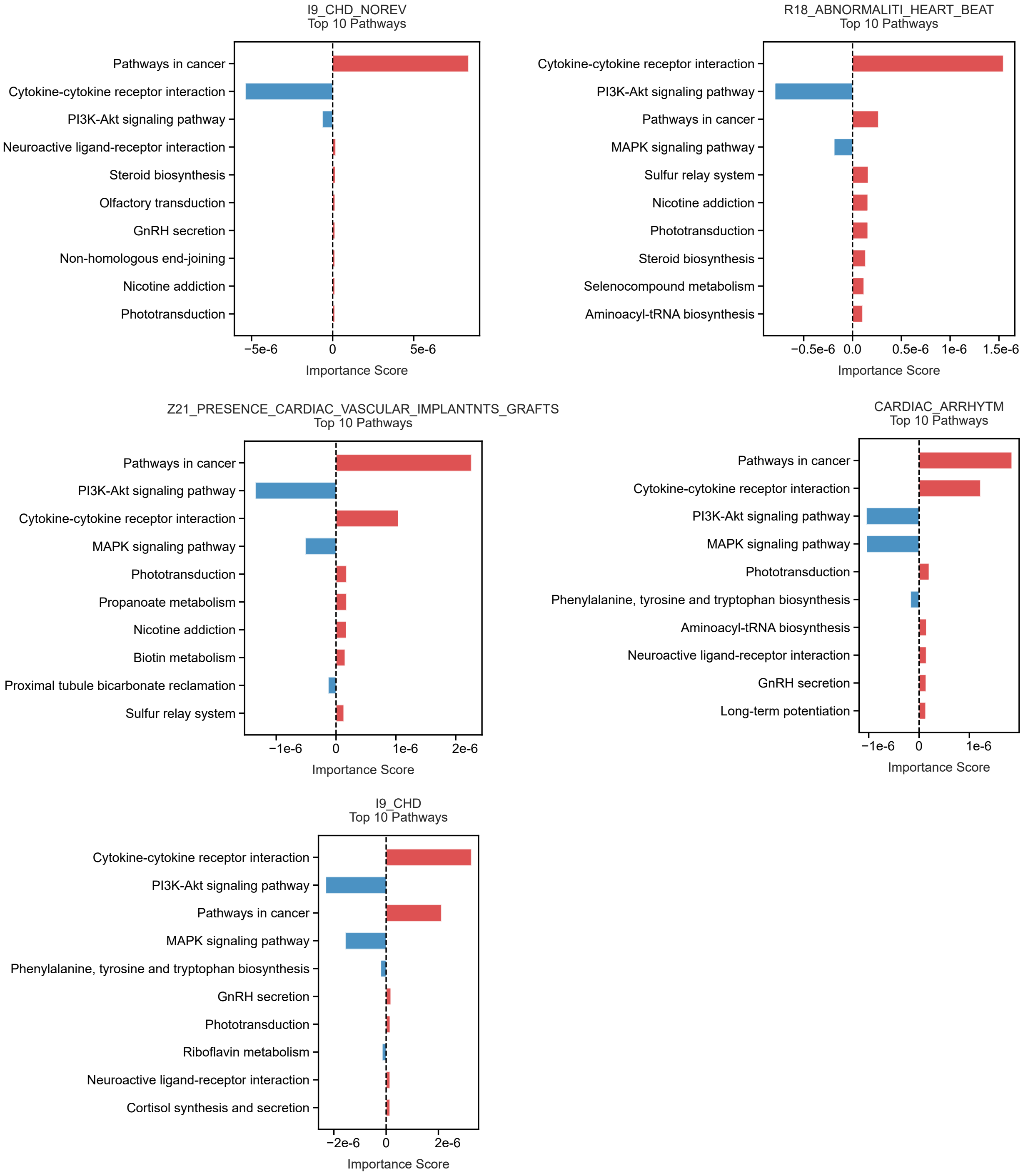

Supplementary Fig. 4: Top-10 biological pathways identified by ProMeta as most predictive for five representative cardiovascular conditions. Pathways are ranked by their aggregated importance score, derived by summing the absolute gradients of constituent proteins.

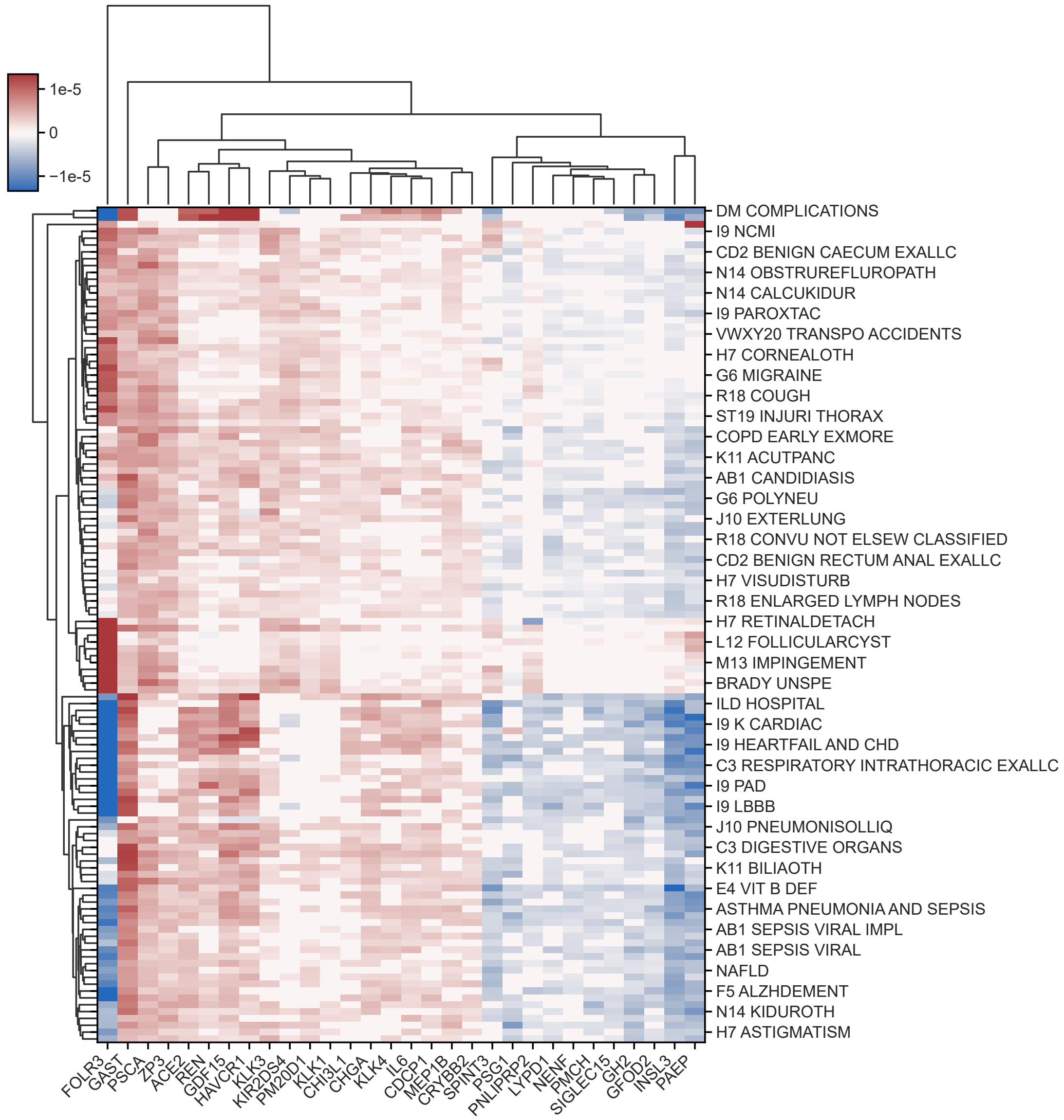

Supplementary Fig. 5: High-frequency protein biomarkers identified by ProMeta for disease prediction. Hierarchical clustering of these top 30 proteins across diverse disease endpoints. The heatmap visualizes the gradient-based importance scores (red: positive association; blue: negative association).

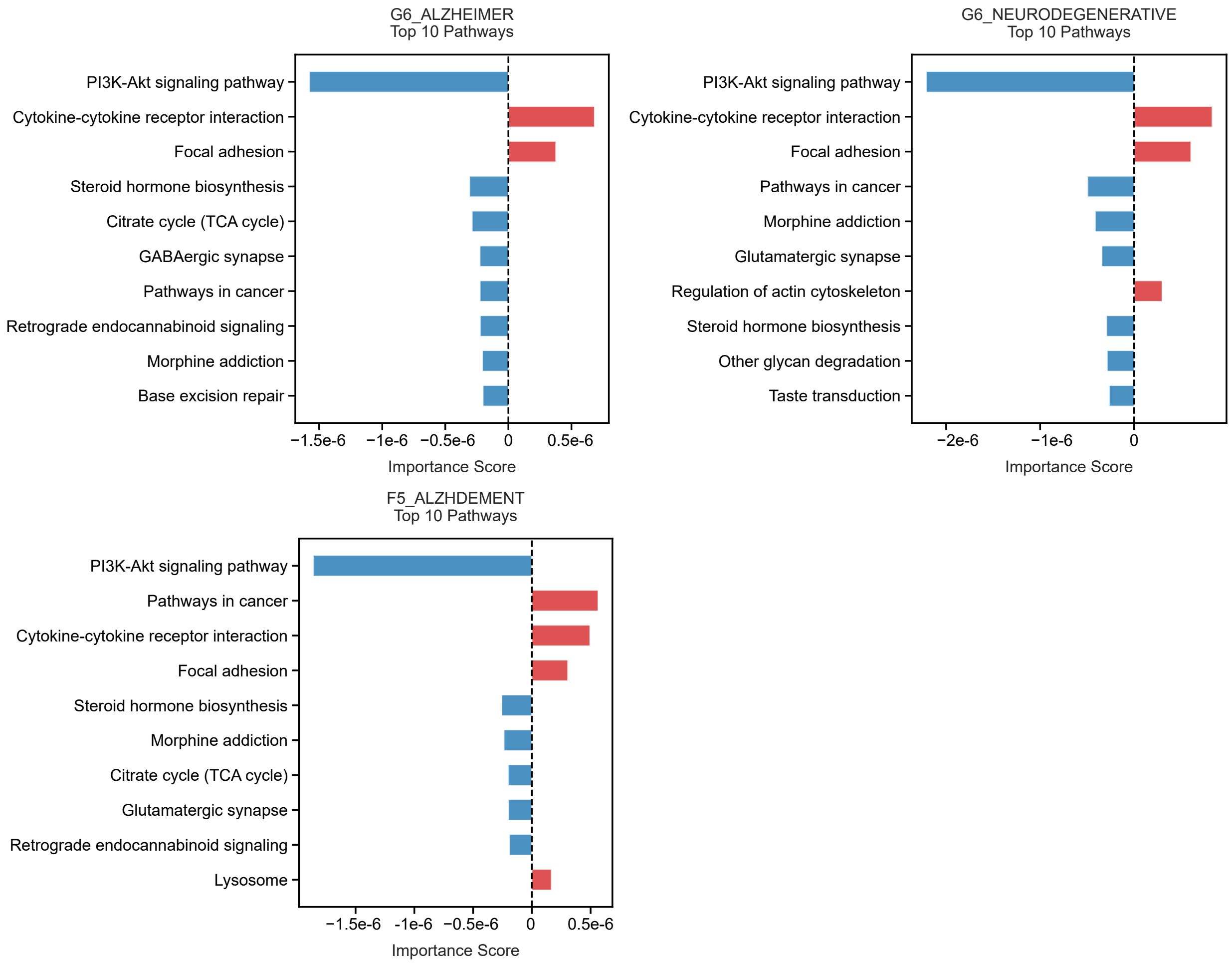

Supplementary Fig. 6: Top-10 biological pathways identified by ProMeta as most predictive for three representative neurodegenerative disorders. Pathways are ranked by their aggregated importance score, derived by summing the absolute gradients of constituent proteins.

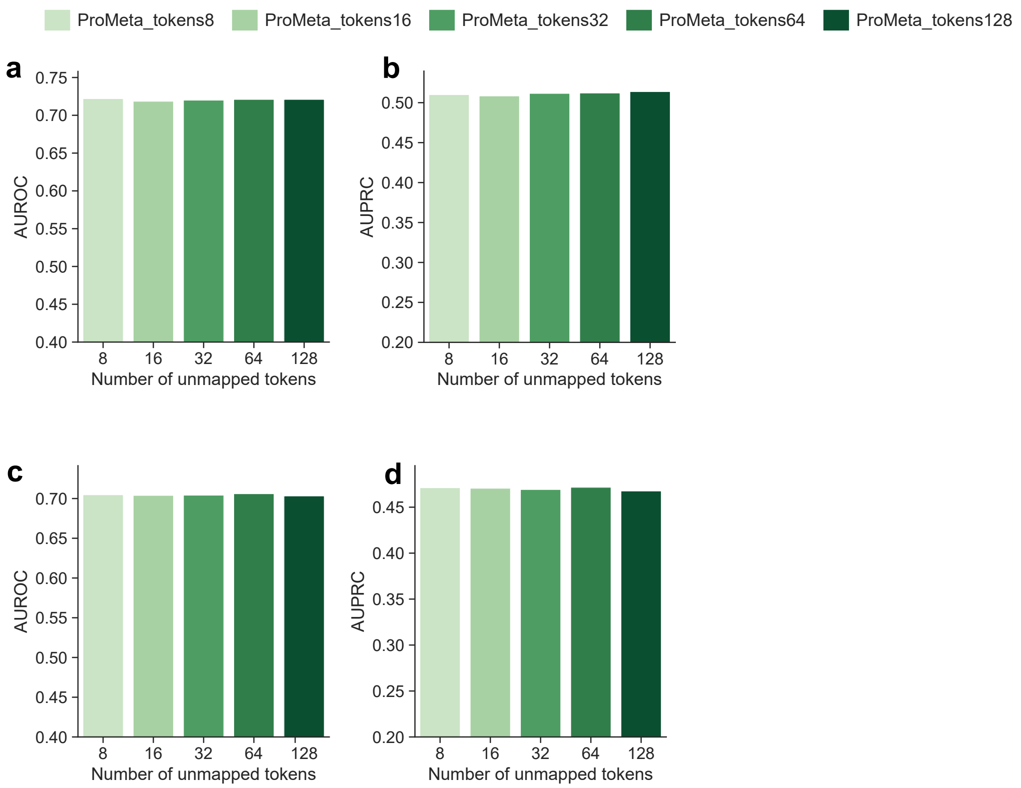

Supplementary Fig. 7: Effect of auxiliary token quantity on predictive accuracy. Performance of ProMeta (AUROC and AUPRC) for (a–b) diagnosis and (c–d) prediction tasks with different token numbers. All evaluations were conducted under the 32-shot setting (random seed 42).

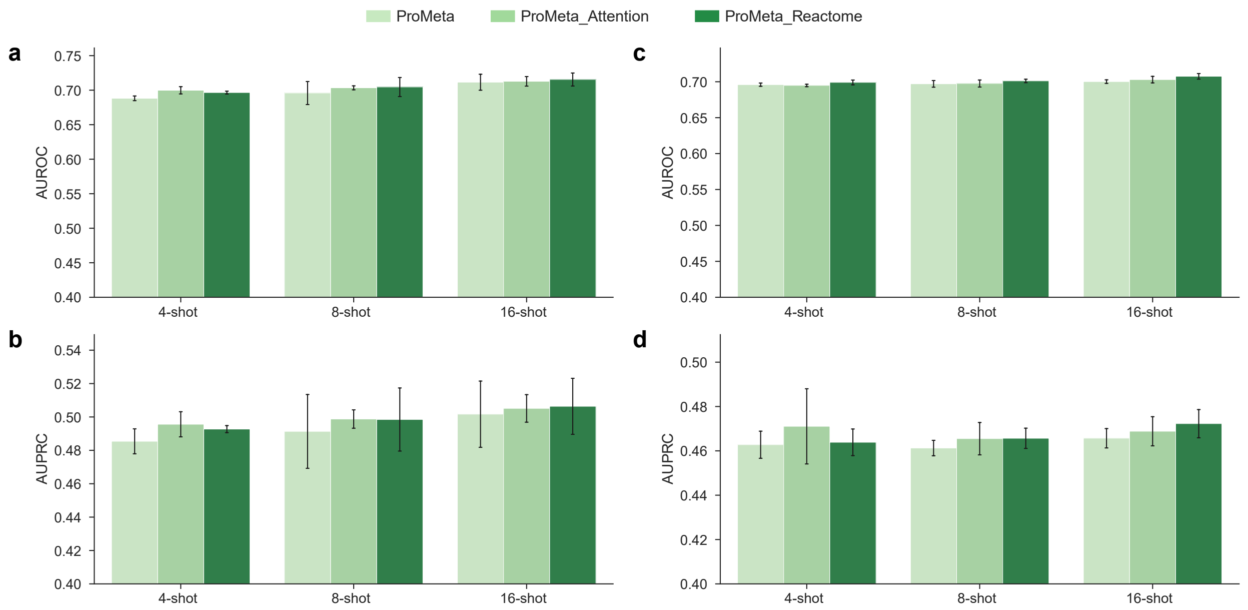

Supplementary Fig. 8: Sensitivity analysis of ProMeta across different biological priors and aggregation mechanisms. (a) AUROC and (b) AUPRC scores for disease diagnosis, and (c) AUROC and (d) AUPRC scores for disease prediction. All models were compared across 4-shot, 8-shot, and 16-shot settings.
